# Efficacy of Presurgical Short-Term Endocrine Therapy During the Waiting Period for Surgery in Postmenopausal Hormone Receptor-Positive Breast Cancer

**DOI:** 10.1155/tbj/9976413

**Published:** 2025-05-22

**Authors:** Yuka Maeda, Ayana Sato, Akiko Matsumoto, Tatsuhiko Ikeda, Hiromitsu Jinno

**Affiliations:** Department of Surgery, School of Medicine, Teikyo University, Tokyo, Japan

**Keywords:** aromatase inhibitors, breast cancer, hormone receptor-positive, neoadjuvant therapy

## Abstract

**Background:** Although presurgical endocrine therapy has been used to enhance the rate of breast cancer conservation, its prognostic relevance is unknown. The search for a valid prognostic factor equivalent to pathological complete response in presurgical chemotherapy remains a challenge in presurgical endocrine therapy. This study investigated the efficacy of presurgical short-term endocrine therapy (preSTE) and assessed prognostic factors, including the preoperative endocrine prognostic index (PEPI) score.

**Methods:** From October 2012 to November 2021, 269 postmenopausal women diagnosed with hormone receptor-positive (HR+), human epidermal growth factor receptor 2 negative breast cancer underwent endocrine therapy with a nonsteroidal aromatase inhibitor during the presurgical waiting period. The primary endpoint was to assess the changes in tumor size using ultrasonography, and Ki67 expression levels before and after preSTE. The secondary endpoint was the prognosis of patients categorized using the PEPI score.

**Results:** The median age of patients was 68 years (range, 41–89 years). The median tumor size was 1.65 cm (range, 0.4–7.5 cm). The average pretreatment Ki67 expression level was 10% (range, 0%–90%). The median duration of endocrine therapy was 39 days (range, 2–88 days). Tumor diameter and Ki67 expression levels were significantly decreased to 1.43 cm (range, 0.45–5.83 cm) and 3.0% (range, 0%–85%) after preSTE, respectively. After the median observation period of 928 days, patients with PEPI scores ≥ 4 showed worse disease-free survival compared with those with lower PEPI scores. In terms of mortality, patients with PEPI score ≥ 4 had worse overall survival than did patients with lower PEPI scores.

**Conclusions:** Endocrine therapy during the waiting period for surgery might be effective in reducing tumor size, and the Ki67 expression level and PEPI score might be useful in predicting the prognosis of patients with postmenopausal HR+ breast cancer.

## 1. Introduction

Breast cancer is the most common type of cancer and the leading cause of cancer-related deaths among women worldwide. Hormone receptor-positive (HR+) tumors are the most common form of this disease, with more than 70% of breast cancers expressing these receptors.

In clinical research and routine practice, presurgical endocrine therapy is considered a valid treatment option for HR +/human epidermal growth factor receptor 2 negative (HER2−) patients. Presurgical endocrine therapy for patients with HR+/HER2− breast cancer is expected to downstage tumors. In addition to enabling less extensive surgery, it serves as a scientific platform for obtaining information on tumor resistance and potential biomarkers [1].

Presurgical chemotherapy studies have firmly established that pathological complete response (pCR) is a strong predictor of survival in patients with HR + HER2− breast cancer. However, a pCR response to presurgical endocrine therapy is very rare. In three large studies on letrozole, the pCR rate was less than 1% [2–4]. Hence, the preoperative endocrine prognostic index (PEPI) was developed using a multivariable model [5]. The PEPI score incorporates pathological tumor size, lymph node metastasis, estrogen receptor (ER) expression, and Ki67 expression level after presurgical endocrine therapy and has demonstrated its validity in estimating recurrence-free survival (RFS) in patients undergoing presurgical endocrine therapy.

In this study, we investigated the efficacy of presurgical short-term endocrine therapy (preSTE) during the waiting period for surgery. Perioperative endocrine therapy for 14 days had no significant improvement on long-term outcome in POETIC trial [6]. It also seems unlikely to reduce the tumor burden by preSTE because preoperative endocrine therapy usually needs 4–6 months of treatment duration. The advantage of preSTE utilizing the waiting duration for surgery is the relief of patients because systemic treatment could begin immediately. Although PEPI score was originally developed based on 4 months of preoperative endocrine therapy, POETIC trial demonstrated that even just two weeks of presurgical endocrine treatment allows for the evaluation of endocrine sensitivity or resistance by measuring Ki67 levels, providing a potential tool for personalized treatment. Therefore, the value of PEPI score as the prognostic factor was evaluated in this study.

## 2. Methods

A total of 336 postmenopausal women with stage I–III, HR+/HER2− breast cancer was treated with endocrine therapy using a nonsteroidal aromatase inhibitor during the preoperative waiting period between October 2012 and November 2021. Among these, 67 were excluded owing to missing preoperative ultrasound data, and 269 were included in the analysis. The patients were orally administered either letrozole (2.5 mg) or anastrozole (1 mg) once daily. Pathological samples were obtained from needle biopsies before treatment initiation and from resected specimens at the time of surgery. ER and progesterone receptor (PgR) expression was evaluated via immunohistochemistry, and positivity was defined as nuclear staining observed in at least 1% of the cells [7, 8]. Ki67 expression levels were evaluated by immunohistochemistry and determined as the mean expression in the whole tumor area [9]. The PEPI score was calculated as described by Ellis et al. [5].

The primary endpoint was the change in tumor size, as measured using ultrasound, and ER and PgR, Ki67 expression levels before and after preSTE. The secondary endpoint was the patient prognosis, divided by the PEPI score. ER and PgR statuses were evaluated using the Allred score. The scores were expressed as mean ± standard deviation (SD) and compared by *t*-test. The mean tumor size and Ki67 expression levels were compared using the *t*-test. Disease-FS (DFS) and overall survival (OS) were estimated using the Kaplan–Meier method and the log-rank test. A *p* value less than or equal to 0.05 was considered statistically significant, and all statistical tests were two-sided. All statistical analyses were conducted using SPSS ver. 24.0 (IBM Co., Armonk, NY, USA).

## 3. Results

The median patient age was 68 years (range, 41–89 years). Median tumor size was 1.65 cm (0.4 to 7.5 cm). A total of 252 (93.7%) patients had no lymph node metastasis (Table 1). In histological classification, 85% of cases had invasive ductal carcinoma. Approximately 70% of cases had nuclear grade 1. Positivity rates for ER and PgR were 100% and 86%, respectively. The average pretreatment Ki67 expression level was 10% (range, 0%–90%). Among the 269 patients, 92 (34.2%) and 177 (65.8%) patients had anastrozole and letrozole, respectively. Baseline characteristics were well balanced between the anastrozole and letrozole group.

The median preSTE duration was 39 days (range, 2–88 days). A total of 177 (65.8%) patients were subjected to lumpectomy, and sentinel lymph node biopsy was carried out in 241 (89.6%) patients.

Tumor diameter significantly decreased from 1.65 cm to 1.43 cm (*p*=0.01), and Ki67 expression levels significantly decreased from 10% to 3% after preSTE (Table 2).

No pCR was observed in any patient (Table 3). ypT1 tumors were found in 195 (72.5%) patients, and ypN0 status was noted in 219 (81.4%) patients. Regarding the PEPI score, 83 (30.9%) patients had a score- of 0, and 39 (14.5%) patients had a score- of ≥ 4.

**Figure 1:**
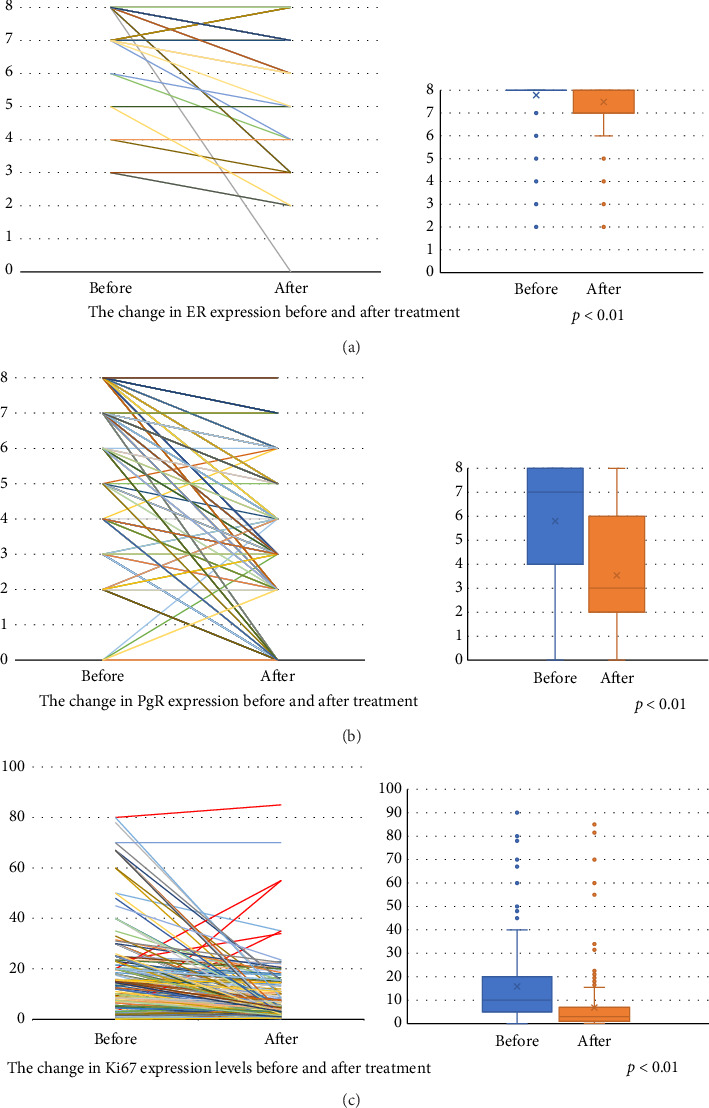
(a) The change in ER expression before and after treatment. (b) The change in PgR expression before and after treatment. (c) The change in Ki67 expression levels before and after treatment.

ER and PgR statuses were negative in 1.86% and 23% of the patients after preSTE, respectively. The expression levels of ER and PgR significantly decreased following preSTE (Figures 1(a) and 1(b)). Although post-treatment Ki67 expression levels decreased in most patients, five patients exhibited a marked increase in Ki67 expression levels (Figure 1(c)).

**Figure 2:**
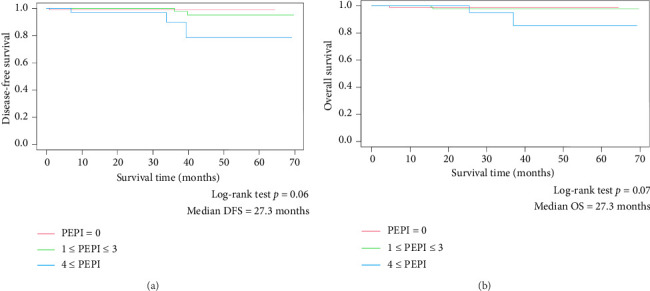
(a) Disease-free survival in patients. (b) Overall survival in patients.

Patients with scores≥ 4 exhibited worse DFS compared with those with scores 0 and 1–3 (*p*=0.06; Figure 2(a)). In terms of mortality, patients with scores≥ 4 had worse OS than patients with scores 0 and 1–3 (*p*=0.07; Figure 2(b)). Although there was no significant difference in DFS and OS according to the PEPI score, patients with lower PEPI scores showed longer DFS and OS.

In terms of recurrent cases by PEPI score, three cases with scores ≥ 4 were due to liver, lung, and pleural metastasis in one case each. Two cases with scores of 1–3 were due to local recurrence and liver metastasis in one case each, and one case with scores of 1–3 was due to bone metastasis. In terms of mortality by PEPI score, two cases with scores ≥ 4 were breast cancer-related deaths due to liver or pleural metastases, whereas three cases with scores 0–3 resulted in deaths due to other diseases. Multivariate analysis adjusted for tumor size, lymph node status, and Ki67 showed that PEPI score was not an independent factor for survival (Data Supplement Tables S1 and S2).

## 4. Discussion

In this study, presurgical endocrine therapy was administered during the waiting period for surgery, for a median duration of 39 days. Despite the short treatment duration, significant reductions in tumor size and Ki67 expression were observed. Although there was no significant difference in DFS and OS according to the PEPI score, patients with low PEPI scores showed longer DFS and OS.

In our study, the clinical response rate to ultrasound was 14.5%, with no CR. Conversely, the response rate in the IMPACT trial was 24%, also with no CRs. The lower response rate in our study could be attributed to the shorter median treatment duration compared to that in the IMPACT study (39 vs. 84 days).

Endocrine therapy induces cell-cycle arrest, and the tumor proliferation may serve as a surrogate marker of treatment effectiveness. The post-treatment median expression of Ki67 expression levels was significantly reduced from 10% to 3% in our study. Regarding the extent of Ki67 expression suppression that correlates with prognosis, existing predictive models suggest that a post-treatment Ki67 threshold of around 3% is associated with a favorable prognosis. The POETIC trial identified that patients with a post-treatment Ki67 below 10% had a lower recurrence risk, while the P024 trial incorporated post-treatment Ki67 into the PEPI, where a Ki67 expression level below 2.7% was associated with a PEPI score of 0, indicating an extremely low relapse risk [6]. The IMPACT trial also demonstrated that tumor Ki67 expression levels after 2 weeks of endocrine therapy were significantly associated with RFS [10]. The WSG-ADAPT-HR+/HER2− trial found that a reduction in Ki67 expression levels after 3-week preoperative endocrine therapy was associated with better outcomes even in patients with intermediate recurrence score [11]. ADAPT combined Ki67 suppression with genomic assays (Oncotype DX Recurrence Score) to guide chemotherapy omission. Patients with RS < 26 and post-treatment Ki67 ≤ 10% had favorable outcomes with endocrine therapy alone, supporting Ki67 as a marker for chemotherapy omission in certain high-risk luminal breast cancer patients. Our observed post-treatment Ki67 median of 3% closely aligns with these predictive models, suggesting a potential association with improved long-term outcomes. These findings reinforce the prognostic relevance of Ki67 suppression and support the utility of post-treatment Ki67 assessment in guiding clinical decisions regarding adjuvant therapy. The mean reduction in Ki67 expression levels with aromatase inhibitors was 87% in the P024 trial, 81.6% in the IMPACT trial, 78% in the ACOSOG Z1031 trial, and 70% in this study. Dieci et al. found that the extent of Ki67 suppression with endocrine therapy was dependent on tumor-infiltrating lymphocytes [12]. In five (1.86%) cases, there was an increase in Ki67 expression levels in post-treatment surgical specimens (Figure 1(c)). Other comparable clinical trials reported that 3.4% to 7.1% of the patients showed an increase in Ki67 expression levels [1, 13].

However, the optimal duration of presurgical endocrine therapy is yet to be determined. In this study which was conducted during the presurgical waiting period, the treatment duration varied from 2 to 88 days, with a median duration of 39 days. The duration of presurgical endocrine treatment in the P024 and IMPACT trials was 4 and 3 months, respectively. However, clinical studies demonstrated that a longer treatment duration may be necessary to achieve efficacy [14–17]. Clinical trials that aimed to identify the optimal treatment duration for achieving maximum efficacy compared 4, 8, and 12 months of letrozole treatment, with pCR rates of 2.5%, 5.0%, and 17.5%, respectively (*p* < 0.04). Longer durations of presurgical endocrine treatment resulted in higher pCR rates [18].

The PEPI score is a distinct prognostic tool for ER-positive breast cancer that relies on tumor and lymph node characteristics post-presurgical endocrine therapy. The PEPI score integrates residual disease burden and cell cycle response, providing a straightforward method for de-escalating adjuvant treatment after presurgical aromatase inhibitor administration for patients with a PEPI score of 0 [19]. In our study, the distribution of PEPI scores 0, 1–3, and ≥ 4 were 30.9%, 54.2%, and 14.5%, respectively, compared to 16.8%, 37.8%, and 29.6% in the ACOSOG Z1031 trial. Adjuvant chemotherapy was administered to 15.1%,12%, 3%, and 0% of patients with a PEPI score of 0 in the ACOSOG Z1013 trial, P024 trial, IMPACT trial, and our study, respectively. Patients with PEPI scores 1–3 who received chemotherapy constituted 37%, 22%, and 9.6% in the P024 trial, IMPACT trial, and our study, respectively. Furthermore, patients with PEPI scores ≥ 4 who received chemotherapy were 54%, 35%, and 31% in the P024 trial, IMPACT trial, and our study, respectively [5]. Alternately, 14.1% of our patients with a PEPI score greater than 0 received adjuvant chemotherapy, compared to 47.5% in the ACOSOG Z1031 study. These results suggest that chemotherapy was omitted in our patients owing to the early stage of the disease. The risk of breast cancer recurrence for patients with a PEPI score of 0, relative to those with a PEPI score greater than 0, was 0.27 (*p*=0.014; 95% CI, 0.092 to 0.764) when stratified by cohort and adjuvant chemotherapy use in ACOSOG Z1031 [19]. As validated by the P024 and IMPACT trials, patients with a PEPI score of 0 had an extremely low risk of recurrence of 10% (median follow-up, 62 months) and 3% (median follow-up, 37 months), respectively [5]. In ACOSOG Z1031, the relapse risk of patients with a PEPI score of 0 was only 3.6% without chemotherapy, supporting the use of adjuvant endocrine monotherapy in this group [19, 20].

In this study, there was no significant difference in DFS and OS according to the PEPI score. The median observation period of 27.3 months is too short to evaluate the prognosis of patients with HR+ breast cancer. The median duration of administration was 39 days in our study, although the calculation of the PEPI score may require a presurgical endocrine therapy duration of 4 months [5].

This study has several limitations. First, the retrospective study design may have caused a selection bias. Second, this was a single-center study, and the number of participants was small. Third, the observation period of 27.3 months is too short to evaluate the prognosis of patients with HR+ breast cancer. Fourth, the types of aromatase inhibitors were not standardized, although the ACOSOG Z1031 trial compared presurgical exemestane, anastrozole, and letrozole, finding no differences in clinical response, Ki67 expression level reduction, or PEPI score among these drugs [21]. Furthermore, the duration of the administration varied.

## 5. Conclusions

These results suggest that implementing endocrine therapy during the presurgical waiting period may effectively reduce tumor size and Ki67 expression levels and that the PEPI score may serve as a valuable predictor for the prognosis of postmenopausal women with HR+ breast cancer.

## Figures and Tables

**Table 1:** Patient and treatment characteristics.

*n* = 269	
Age, median (range)	68 (41–89)
Primary tumor size, median, cm (range)	1.65 (0.4–7.5)
Clinical T stage	
T1	135 (50.2)
T2	121 (45.0)
T3	4 (1.5)
T4	9 (3.3)
Clinical N stage	
N0	252 (93.7)
N1	16 (5.9)
N2	1 (0.4)
Staging group	
I	132 (49.1)
II	124 (46.1)
III	9 (3.3)
Histological type	
Invasive ductal carcinoma	229 (85.1)
Invasive lobular carcinoma	14 (5.2)
Mucinous carcinoma	20 (7.4)
Other	6 (2.2)
Nuclear grade	
1	190 (70.6)
2	42 (15.6)
3	29 (10.8)
Unknown	8 (3.0)
ER (Allred score)	
0–2	1 (0.4)
3–8	268 (99.6)
PgR (Allred score)	
0–2	36 (13.4)
3–8	232 (86.2)
Unknown	1 (0.4)
Ki67 expression	
0–2.7	24 (8.9)
> 2.7–7.3	73 (27.1)
> 7.3–19.7	87 (32.3)
> 19.7–53.1	68 (25.3)
> 53.1	15 (5.6)
Unknown	2 (0.7)
Presurgical endocrine therapy	
Anastrozole	92 (34.2)
Letrozole	177 (65.8)
Duration of therapy, median (days)	39 (2–88)

*Note:* Data are expressed as *n* (%) unless otherwise specified.

Abbreviations: ER = estrogen receptor, PR = progesterone receptor.

**Table 2:** Change of tumor size and Ki67 expression levels.

	Pretreatment	Post-treatment	*p* value
Tumor size (median (cm (range)))	1.65 (0.4–7.5)	1.43 (0.45–5.83)	*p*=0.01
Ki67 expression levels (median (% (range)))	10 (0–90)	3 (0–85)	*p*<0.01

**Table 3:** Pathological outcomes after presurgical endocrine therapy.

*n* = 269	
Tumor and axillary staging	
Pathological complete response^∗^	0
ypT1ypN0	172 (64.0)
ypT1ypN1	21 (7.8)
ypT1ypN2	1 (0.4)
ypT1ypN3	1 (0.4)
ypT2ypN0	36 (13.4)
ypT2ypN1	14 (5.2)
ypT2ypN2	3 (1.1)
ypT3ypN0	2 (0.7)
ypT3ypN1	2 (0.7)
ypT4ypN0	9 (3.3)
ypT4ypN1	6 (2.2)
ypT4ypN3	1 (0.4)
Unknown	1 (0.4)
PEPI score	
0	83 (30.9)
1–3	146 (54.2)
≥ 4	39 (14.5)
Unknown	1 (0.4)
ER (Allred score)	
0–2	3 (1.1)
3–8	266 (98.9)
PgR (Allred score)	
0–2	90 (33.5)
3–8	177 (65.8)
Unknown	2 (0.7)
Ki67 expression	
0–2.7	109 (40.5)
> 2.7–7.3	94 (34.9)
> 7.3–19.7	43 (16.0)
> 19.7–53.1	16 (5.9)
> 53.1	6 (2.2)
Unknown	1 (0.4)
Ki67 expression (median (range))	3.0 (0–85)

*Note:* Data are expressed as *n* (%) unless otherwise specified.

Abbreviations: PEPI = Preoperative endocrine prognostic index.

^∗^Pathological complete response was defined as the absence of invasive disease in the breast and axilla (ypT0/is ypN0).

## Data Availability

The data that support the findings of this study are available from the corresponding author upon reasonable request.

## Nomenclature

CR: Complete response
DFS: Disease-free survival
ER: Estrogen receptor
HER2: Human epidermal growth factor receptor 2 negative
HR+: Hormone receptor-positive
ITSA: Interrupted time series analysis
OS: Overall survival
PEPI: Preoperative endocrine prognostic index
PgR: Progesterone receptor
pCR: Pathological complete response
RFS: Recurrence-free survival.

## Supporting Information

Table S1: Multivariate analysis of disease-free survival (DFS).

Table S2: Multivariate analysis of overall survival (OS).
