# Supplementary material for: Efficacy of Presurgical Short-Term Endocrine Therapy During the Waiting Period for Surgery in Postmenopausal Hormone Receptor-Positive Breast Cancer
Source: Breast J. 2025 May 22;2025:9976413. doi: 10.1155/tbj/9976413 (PMC12122150; doi:10.1155/tbj/9976413)
Supplement: Supporting Information — Additional supporting information can be found online in the Supporting Information section. [file 9976413.f1.zip › Supplement Table S1 Multivariate Analysis of Disease-free survival (DFS).docx]

**Supplementary Table S1** Multivariate Analysis of Disease-free survival (DFS)

| Variable | HR | 95% CI Lower | 95% CI Upper | p-value |
| --- | --- | --- | --- | --- |
| Age | 1.01 | 0.93 | 1.09 | 0.87 |
| pT | 1.03 | 0.40 | 2.61 | 0.96 |
| pN | 2.12 | 0.46 | 9.73 | 0.33 |
| Ki67 | 0.85 | 0.59 | 1.21 | 0.35 |
| PEPI score | 1.26 | 0.50 | 3.16 | 0.62 |
