# Supplementary material for: Efficacy of Presurgical Short-Term Endocrine Therapy During the Waiting Period for Surgery in Postmenopausal Hormone Receptor-Positive Breast Cancer
Source: Breast J. 2025 May 22;2025:9976413. doi: 10.1155/tbj/9976413 (PMC12122150; doi:10.1155/tbj/9976413)
Supplement: Supporting Information — Additional supporting information can be found online in the Supporting Information section. [file 9976413.f1.zip › Supplement Table S2 Multivariate Analysis of Overall Survival (OS).docx]

**Supplementary Table S2** Multivariate Analysis of Overall Survival (OS)

| Variable | HR | 95% CI Lower | 95% CI Upper | p-value |
| --- | --- | --- | --- | --- |
| Age | 1.06 | 0.96 | 1.16 | 0.27 |
| pT | 1.51 | 0.58 | 3.94 | 0.40 |
| pN | 4.04 | 1.01 | 16.18 | 0.05 |
| Ki67 | 1.02 | 0.91 | 1.13 | 0.79 |
| PEPI score | 0.78 | 0.34 | 1.79 | 0.56 |
